# Beyond the 510(k): The regulation of novel moderate-risk medical devices, intellectual property considerations, and innovation incentives in the FDA’s De Novo pathway

**DOI:** 10.1038/s41746-024-01021-y

**Published:** 2024-02-08

**Authors:** Mateo Aboy, Cristina Crespo, Ariel Stern

**Affiliations:** 1 Centre for Law, Medicine, and Life Sciences (LML), Faculty of Law, University of Cambridge, Cambridge, UK; 2 Harvard Business School and Harvard-MIT Center for Regulatory Science, Boston, MA USA; 3 Hasso-Plattner-Institut für Digital Engineering, Potsdam, Germany

**Keywords:** Technology, Intellectual-property rights

## Abstract

Moderate-risk medical devices constitute 99% of those that have been regulated by the U.S. Food and Drug Administration (FDA) since it gained authority to regulate medical technology nearly five decades ago. This article presents an analysis of the interaction between the 510(k) process —the historically dominant path to market for most medical devices— and the De Novo pathway, a more recent alternative that targets more novel devices, including those involving new technologies, diagnostics, hardware, and software. The De Novo pathway holds significant potential for innovators seeking to define new categories of medical devices, as it represents a less burdensome approach than would have otherwise been needed historically. Moreover, it supports the FDA in its effort to modernize the long-established 510(k) pathway by promoting the availability of up-to-date device “predicates” upon which subsequent device applications can be based, reflecting positive spillovers that are likely to encourage manufacturers to adopt current state-of-the-art technologies and modern standards of safety and effectiveness. We analyze the of characteristics all the De Novo classification requests to date, including the submission type, trends, FDA review times, and device types. After characterizing how the De Novo process has been used over time, we discuss its unique challenges and opportunities with respect to medical device software and AI-enabled devices, including considerations for intellectual property, innovation, and competition economics.

## Introduction

Medical devices have played a critical role in raising the standards of healthcare delivery. The COVID-19 pandemic underscored clinical medicine’s dependence on devices ranging from diagnostic test kits to pulse oximeters, physiologic monitors, and ventilators. Medical devices are now essential for effective disease prevention, diagnosis, treatment and rehabilitation. The global medical device market is expected to grow from $471 billion in 2020 to $623 billion in 2026^1^. A great deal of the innovation in medical devices currently comes from software (Box 1).

The U.S. Food and Drug Administration (FDA) Center for Devices and Radiological Health (CDRH) regulates medical devices in the United States. Before medical devices can be legally marketed, the firm seeking to commercialize a new device must pursue one of the available FDA regulatory pathways to demonstrate that the device is safe and effective.

The FDA’s 510(k) pathway has been the most widely employed regulatory pathway since the enactment of the 1976 Medical Device Amendments (MDA) to the Federal Food, Drug, and Cosmetic (FD&C) Act, which first gave the FDA authority to regulate medical devices. It is a premarket submission intended for moderate-risk medical devices. Of the >155,000 devices approved or cleared by the FDA since 1976, ~99% used the 510(k) pathway^2^.

A 510(k) is a premarket submission made to the FDA “to demonstrate that the device to be marketed is as safe and effective, that is, substantially equivalent, to a legally marketed device” (i.e., the *predicate* device)^3^. The initial list of predicates were the devices that were already legally marketed in the US before the MDA’s passage in 1976. These “preamendment devices” were grandfathered and established the generic device categories and predicates for the 510(k) pathway. The FDA has established classifications for ~1700 different generic types of devices, grouped into 16 medical specialties known as device classification panels^4^.

In the United States, medical devices are classified into one of three classes based on risk (Box 2). Regulatory controls increase from Class I (low risk) to Class III (high risk). This classification determines the requirements a device must meet prior to market introduction. In particular, non-exempted class I (low risk) and class II (moderate risk) devices for which a predicate device exists can rely on the 510(k) premarket notification pathway—resulting in a medical device *clearance* to market—instead of the significantly more onerous Premarket Approval (PMA) application pathway used primarily for Class III (high risk) devices. Accordingly, the 510(k) program became the preferred and dominant pathway for medical device manufacturers introducing low and moderate risk devices. Critics have long cited the shortcomings of the 510(k) process^5^ and researchers have illustrated that its lack of specificity allows manufacturers to cite predicate devices with a questionable safety record, to the detriment of future device safety^6^. In 2018, the FDA published an updated *Medical Device Safety Action Plan*^7^ and in September of 2023, CDRH released a trio of draft guidance documents that, in their final form, will influence the use predicate devices and the generation of clinical evidence going forward^8–10^.

A successful 510(k) submission requires the applicant to demonstrate “substantial equivalence” (SE) between the new device and at least one legally marketed “predicate” device(s). This is determined based on satisfying the 510(k) inquiries during the substantial equivalence evaluation by the FDA, including: (1) “Do the devices have the same *intended use*?” and (2) “Do the devices have the same *technological characteristics*?” The new device is compared against the *predicate* device(s) in terms of their respective characteristics, including design, principles of operation, materials, and energy use. Any differences between the devices cannot raise “different questions of safety and effectiveness”^11^. That said, devices may use multiple predicates—indeed a recent comprehensive study^6^ found that the average number of predicates per 510(k) submission was 2.6. Historically, a regulatory determination of “Not Substantially Equivalent” (NSE) prompted an automatic classification as a class III (high risk) device and the need for a PMA application, even for low and moderate risk devices.

This situation incentivized applicants to characterize their new medical devices as having the “same intended use” and “same technological characteristics” as a predicate device, independently of the degree of novelty. Manufacturers of low and moderate risk devices have needed to be cautious of introducing significant innovations, as these could have resulted in an NSE determination, and a significantly more demanding regulatory approval process (PMA) intended for high-risk devices, thus potentially hindering innovation.

The De Novo classification process was originally created by Congress in the Food and Drug Administration Modernization Act of 1997 (FDAMA) with the goal of fostering the development of innovative medical devices by providing an intermediate pathway between a 510(k) submission and a PMA application. The De Novo process provides a regulatory pathway to classify novel medical devices for which there is no legally marketed predicate device (Box 2). Via a successful De Novo classification, (1) the new device is classified as a class I or class II device based on a risk-based classification process to determine whether general controls alone (class I) or the combination of general and special controls (class II) provide reasonable assurance of safety and effectiveness for the intended use, (2) a new regulatory category (product code) is created for the specific type of medical device which specifies the class and controls required to ensure safety and effectiveness, and (3) the novel medical device becomes the first predicate under this new regulatory category that can be used as the basis for future 510(k) submissions.

**Fig. 1:**
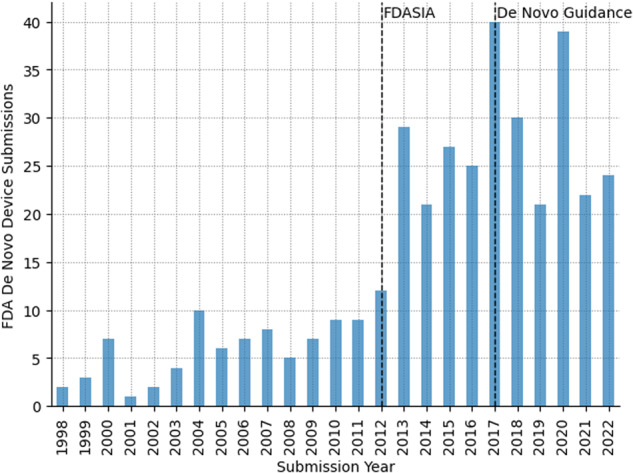
Number of De Novo classification requests over time.

During the first 15 years after its creation the De Novo pathway was rarely used (Fig. 1). This was in part due to the fact that it was not available to manufacturers directly; rather, applicants were required to first submit a 510(k) based on the closest available predicate device. Only if the 510(k) resulted in a NSE determination was a De Novo request and subsequent classification possible. The process changed in 2012 with the enactment of the Food and Drug Administration Safety and Innovation Act (FDASIA), which gave authority to the FDA to review “direct” De Novo submissions. However, no FDA guidance on submission or acceptance was available for several more years, resulting in lingering uncertainty for medical device innovators wishing to use the De Novo pathway.

On October 30, 2017 the FDA published a regulatory guidance document on the “De Novo Classification Process”^12^ to provide clarity on the process for submission and review of De Novo requests. On September 9, 2019, it issued another guidance document on the Acceptance Review for De Novo Classification Requests Guidance^13^ to further support the De Novo process as a pathway to classify novel medical devices without a legally marketed predicate device. This alternative pathway is now available to both (1) applicants receiving a NSE determination (i.e., instead of resulting in an automatic class III classification and associated need for a PMA application), and (2) applicants claiming that there is no legally marketed device upon which to base a determination of SE (without having to first submit a 510(k)). The latter option, in effect, created a third regulatory pathway (direct submission of a De Novo Classification request) for medical device applicants.

There are a number of outstanding questions about the De Novo classification program that are important for assessing its use, function, effectiveness, and potential. This study provides an overview and analysis of the devices classified under Section 513(f)(2)(De Novo) and a discussion of the associated implications. In particular, we analyze regulatory data collected on all the medical devices that came to market via the De Novo pathway between 1997 and 2023 to answer the following ten questions: (1) How frequently has the De Novo process been used?, (2) How has the number of FDA De Novo classification requests changed over time?, (3) What is the proportion of “direct” De Novo requests among all such applications?, (4) What have been typical review times for De Novo classification requests?, (5) Are some types of devices more suitable for the De Novo pathway than others?, (6) Is the De Novo process being used (or used more frequently) as a regulatory pathway for SaMD (Software as a Medical Device—i.e., fully software-based products)?, (7) Under what conditions could a successful De Novo classification facilitate competitors’ entry into the market?; (8) Under what conditions could a successful De Novo classification raise the barriers to entry for competitors’?, (9) Are there unique IP considerations associated with the De Novo process that should be kept in mind by applicants and the FDA, and do they differ for SaMD vs. hardware devices?, and (10) What should we expect regarding future use of the De Novo pathway, and what does this tell us about the potential role of the FDA in medical device innovation?

Box 1 Software as Medical Devices (SaMD) The term “Software as a Medical Device” (SaMD) is defined as software intended to be used for medical purposes, that performs these purposes without being part of a hardware medical device^24^. Thus, SaMDs are capable of running on general purpose computing platforms (e.g., computers, smartphones, watches) to achieve the intended medical purposes, without the need for specialized hardware medical devices^25^. This includes mobile apps for medical purposes running on smartphones or watches, as they meet the SaMD definition and are regulated as SaMDs (see examples in Table 1).

Conversely, software does not meet the definition of SaMD if its intended purpose is to drive a hardware medical device. This is referred to as “Software in a medical device” or software “part of” a medical device. As an example, software required by a hardware medical device to perform the medical device’s intended use is not a SaMD, even if sold separately from the hardware medical device. This includes all software used to “drive and control” hardware medical devices, ranging from embedded software or firmware to the application software needed for the device to perform its intended function.

The use of software as medical devices (SaMDs) has grown rapidly in recent years and the market is expected to reach $86.45 billion in 2027, with an estimated Compound Annual Growth Rate (CAGR) of 21.9%^26^.

Box 2 Device risk classification, regulatory controls, and regulatory submission pathways

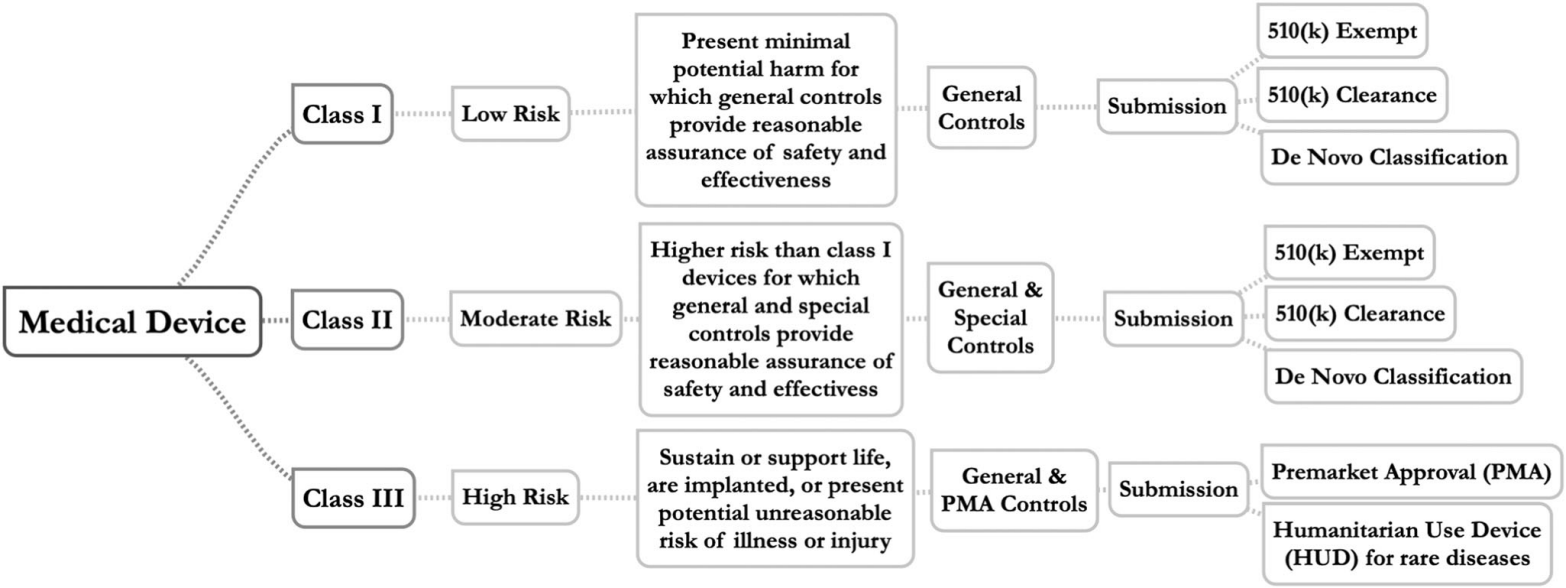

## Results

### How frequently has the De Novo process been used?

There have been a total of 374 De Novo classification requests over the history of the pathway (1997 to August 2023). Since the publication of the FDA’s De Novo *Classification Process* guidance in 2017 there have been between 21 and 40 De Novo classification requests per year (Fig. 1). For comparison, during this period, the FDA cleared an average of 2929 510(k)-track devices, approved an average of 34 PMA-track devices applications, and classified an average of 26 De Novo requests per year, rendering the De Novo pathway about 0.89% the size of the 510(k) pathway and 76% the size of the PMA pathway in recent years^14^.

### How has the number of FDA De Novo classification requests changed over time?

As seen in Fig. 1, there was minimal use of the De Novo classification process over the 15 years following its enactment by Congress in FDAMA (1997), with the number of De Novo submissions *per annum* remaining at or below 10 until 2012 (pre-FDASIA period). In contrast, there was a sustained increase in submissions following the reforms to the De Novo process implemented with FDASIA in 2012, with the number of submissions *per annum* exceeding 20 every year from 2013 to 2022. One of the key reforms introduced by FDASIA was the creation of a “direct” De Novo pathway. As previously noted, prior to FDASIA, a De Novo application was only permitted after an NSE determination in a 510(k) application. The greatest number of De Novo requests (*n* = 40 submissions) was observed in 2017, coinciding with the FDA’s publication of the “De Novo *Classification Process*” guidance document in 2017. This suggests the influence and impact of this particular piece of FDA guidance on industry practices.

### What is the proportion of “direct” De Novo requests among all such applications?

Prior to 2013, all De Novo *classifications* resulted from a failure to obtain 510(k) clearance for the device due to a NSE determination. Since the 2017 publication of the FDA De Novo guidance this trend has almost reversed. From 2017 to August 2023 (*n* = 180), 97.22% of the applications were “direct” De Novo classification requests; the overwhelming majority of De Novo applicants are now opting into this regulatory pathway deliberately.

### What have been typical review times for De Novo classification requests?

The mean decision time for De Novo requests over the period of observation was 338 days (median = 309 days). For comparison, the FDA mean review times were 150 days for 510(k)s and 399 for PMA devices over a similar period of time^2^. Thus, on average, De Novo decision times were 2.3-fold longer than the FDA 510(k) review times and were roughly 15% shorter than contemporaneous PMA review times.

**Fig. 2:**
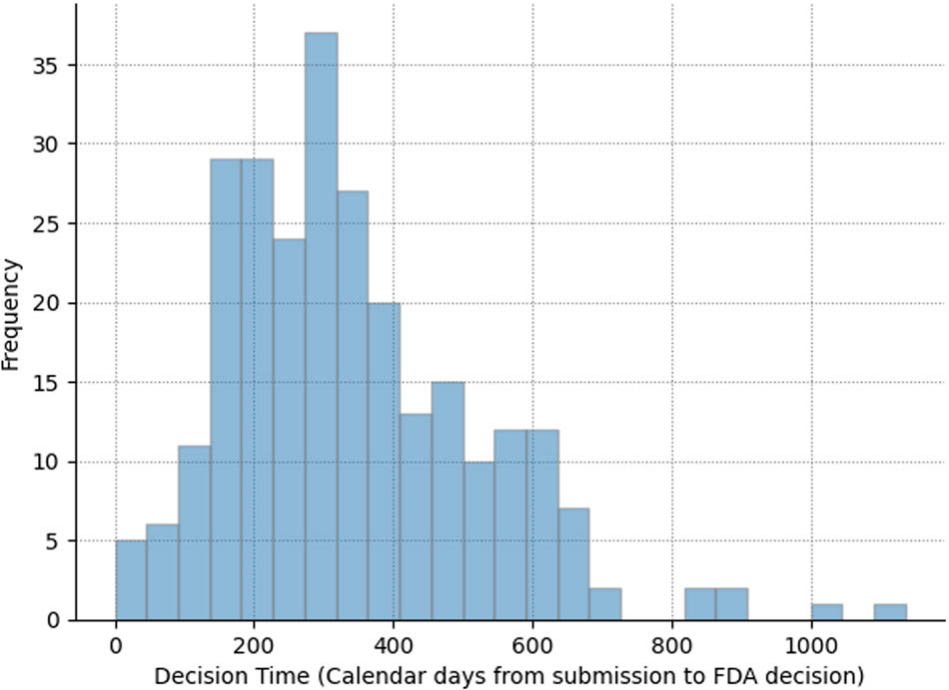
FDA decision times for direct De Novo classification requests submitted from 1998 to 2023.

However, as shown in Fig. 2, De Novo review times varied substantially. Decision times for De Novo submissions ranged from <1 month to over 30 months. The heterogeneity in decision times may not be uniform across product types, and could be due to higher submission rates or fewer resources associated with certain FDA device classification panels for the different medical specialties^4^. Among the fastest De Novo decision times in our sample were Apple’s De Novo requests for the Apple Watch “ECG App” and the “Irregular Rhythm Notification Feature” (28 and 33 days, respectively).

### Are some types of devices more suitable for the De Novo pathway than others?

**Fig. 3:**
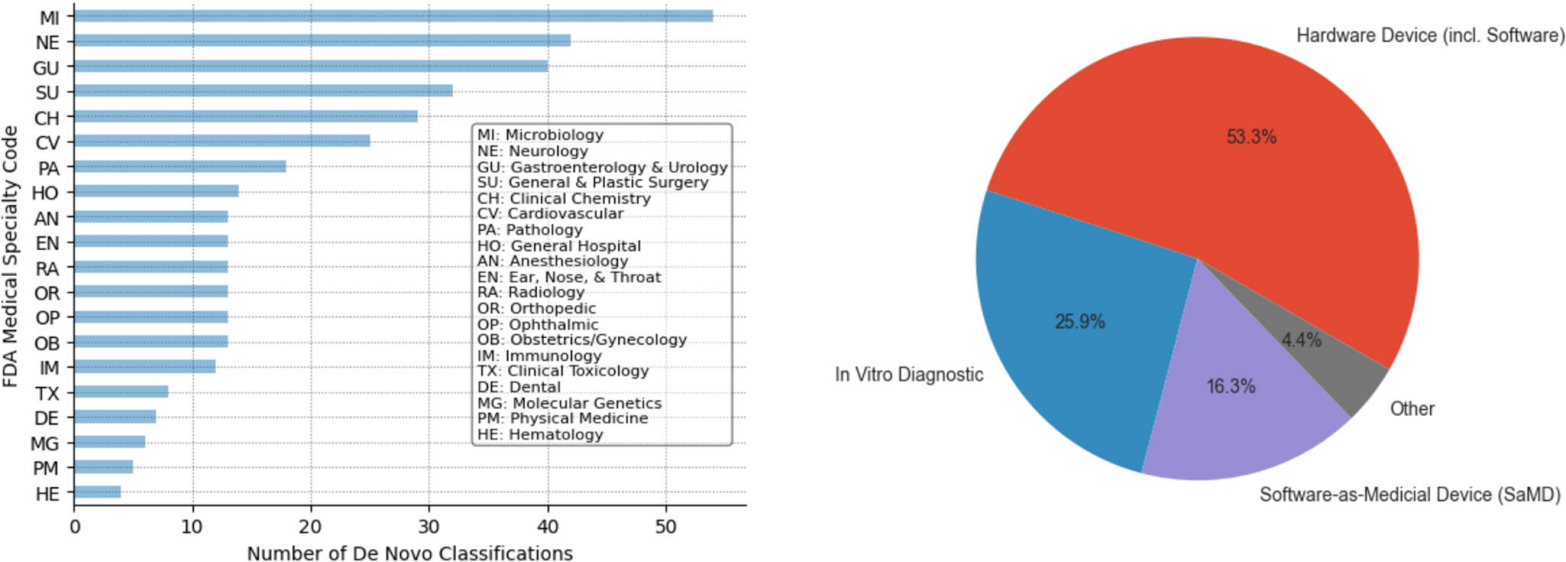
Classification of FDA De Novo requests (1997-2023) by application domain based on the FDA Medical Specialty (left) and by category (2017-2023) of medical device: hardware MD, software (SaMD), in vitro diagnostic (right).

As seen in Fig. 3, since 2017 the De Novo pathway has been used both for hardware (53.3%) and software (16.3%) medical devices, as well as In Vitro Diagnostics (IVDs). IVDs are regulated as medical devices and we find that a significant proportion of FDA De Novo submissions are for IVDs (25.9%). The FDA De Novo process is now the preferred regulatory pathway for novel diagnostics for which there is no predicate device. Notably, these are innovations at the category level that establish new regulatory product types (product codes) for modern diagnostics and, as such, establish the first IVD predicates in these categories.

Figure 3 also highlights that more than half of all De Novo submissions to date are clustered into five primary medical specialty areas (as defined by their respective FDA advisory committees), namely microbiology (14.4%), neurology (11.22%), general and plastic surgery (10.69), gastroenterology/urology (8.5%), clinical chemistry (7.75%) and cardiovascular (6.9%). In contrast, less than 2% of submissions have been in the areas of dental, molecular genetics, physical medicine or haematology.

As a historical example to illustrate the De Novo pathway, the Apple ECG app (DEN180042) did not have a suitable 510(k) predicate device because, *inter alia*, all the potential ECG device predicates were *prescription only (Rx)* and the Apple ECG app was designed and marketed for an over-the-counter (OTC) intended use. Thus, upon its successful De Novo classification request, a new device category (Product Code: QDB) was created for *“Photoplethysmograph analysis software for over-the-counter use”* and added to 21 CFR 870.2790 for *“photoplethysmograph analysis software device for over-the-counter use analyzes photoplethysmograph data and provides information for identifying irregular heart rhythms”*.

This example helps illustrate (1) the De Novo process resulting in the creation of a new product code and regulatory section, (2) the fact that novel OTC digital health products will likely require De Novo classification requests (since the majority of the classical 510(k)’s predicates are prescription only/Rx), and (3) the interaction of De Novo with the 510(k) clearance pathway for follow on devices.

### Is the De Novo process being used (or used more frequently) as a regulatory pathway for SaMD (Software as a Medical Device)?

Although the regulatory development of the common framework and principles for SaMD is fairly recent, Fig. 3 shows that 16.3% of the overall De Novo submissions since 2017 have been for SaMDs. It is important to note that this percentage includes only De Novo requests where software alone is the medical device. Table 1 shows illustrative examples of novel SaMDs, such as the Apple ECG App. These represent cases where medical device innovation was possible due to advances in sensors and algorithms and the associated software running in generally available “off-the-shelf” platforms such as personal computers, smartphones, and smartwatches. It does not include instances of medical device software tied to special purpose hardware, although studies categorizing digital medical devices more broadly have shown that embedded software has also grown substantially in recent years^15^. As digital health technology continues to advance, software is becoming a key part of a large proportion of medical device products. Consequently, although not classified as SaMD, software is an important differentiator in many hardware-based medical devices (roughly half of De Novo hardware medical devices mention a software component), as well as some of the IVDs captured in Fig. 3 (e.g., firmware, advanced algorithms for improved diagnosis, applications for monitoring and analysis).

**Table 1:** Examples of De Novo SaMD devices, including the number, applicant, proprietary device name, prescription (Rx) vs over-the-counter (OTC), and FDA review committee.

K-Number	Applicant	Device name	Rx/OTC	Panel
DEN200069	Cognoa, Inc.	Cognoa ASD Diagnosis Aid	Rx	NE
DEN200019	Oxehealth Limited	Oxehealth Vital Signs	Rx	CV
DEN200029	Mahana Therapeutics, Inc.	Parallel	Rx	GU
DEN190029	Edwards Lifesciences	Acumen Assisted Fluid Management (AFM) Softwar…	Rx	CV
DEN200033	NightWare, Inc	NightWare Kit, Apple iPhone, Apple Watch, Appl…	Rx	NE
DEN200026	Akili Interactive Labs Inc.	EndeavorRx	Rx	NE
DEN190040	Bay Labs, Inc.	Caption Guidance	Rx	RA
DEN190034	Tandem Diabetes Care, Inc.	Control-IQ Technology	Rx	CH
DEN180042	Apple Inc	Irregular Rhythm Notification Feature	OTC	CV
DEN180044	Apple Inc	ECG App	OTC	CV
DEN180005	Imagen Technologies, Inc.	OsteoDetect	Rx	RA

Clearances relying on the Apple ECG App De Novo device as their predicate. These include 510(k) clearances from Apple (*n* = 3), Samsung (*n* = 1), and Fitbit (*n* = 1) now in this product code.

### Under what conditions could a successful De Novo classification facilitate competitors’ entry into the market?

This question represents an important consideration when analyzing the impact of the De Novo regulatory pathway on competition in medical device product markets. At first glance, it may appear that the existence of a successful De Novo classification would lower the barriers to entry for competitors, much in the way that high-risk device categories see faster regulatory approval for follow-on products^16^. The De Novo applicant carries the burden of producing evidence to demonstrate safety and effectiveness for a new medical device category. Following a successful De Novo classification, competitors can then use this device as a predicate and obtain market clearance for their “substantially equivalent” device through the simpler, faster, and cheaper 510(k) application process, without (necessarily) having to provide clinical data. For instance, substantially equivalent devices to the ECG App with the Irregular Rhythm Notification Feature (DEN180042) no longer require a De Novo classification. Our data shows that there have been 5 follow-on 510(k)s.

The interaction between the De Novo classification for the novel device without predicates and the 510(k) for subsequent substantially equivalent devices has similarities with the introduction of a generic drug (i.e., without the need for further clinical trials), except in contrast to a novel branded drug the De Novo applicant does not receive any market exclusivity for their clinical data. In effect, this dynamic—namely a first mover regulatory disadvantage without the benefit of regulatory exclusivity—could discourage De Novo requests. Further, since software is cheaper and faster to develop than hardware, one could imagine that a second- or later-mover advantage would be even clearer for SaMD products. To the extent this is true, the De Novo pathway could therefore have differential effects on competition and market entry in software vs. hardware devices.

Under some conditions successful De Novo classifications can facilitate competitors’ entry into the market, since the newly classified De Novo device can be used as a predicate for substantially equivalent devices. And the 510(k) clearance pathway is generally easier and faster. This is especially the case if the resulting product code for follow-on 510(k) clearances does not require performance standards based on clinical studies because these were either not present as part of the De Novo submission or -if present- the De Novo applicant did not make the case that clinical data was necessary to demonstrate safety and effectiveness. Previous research indicated that ~20% of the De Novo devices were authorized without pivotal studies^17^. In these cases, it is likely that the De Novo applicants are facilitating the competitor’s entry into the market.

### Under what conditions could a successful De Novo classification raise the barriers to entry for competitors’?

It is also possible for De Novo applicants to raise the barriers of entry for subsequent competitors. A De Novo applicant that has accumulated substantial regulatory-grade data through clinical studies can argue to the FDA—as part of the De Novo process—that special controls involving performance standards are needed to ensure the safety and effectiveness of devices in the same (newly-established) category, requiring comparable clinical studies to be conducted in all follow-on 510(k) submissions. This is already the practice for some types of 510(k)s, which require clinical studies to document performance standards and will be further clarified through the finalization of draft guidance on *Recommendations for the Use of Clinical Data in Premarket Notification [510(k)] Submissions*^18^. For example, 510(k) submissions for noninvasive blood pressure measurement devices (Product Code: DXN, Regulatory Number 870.1130) already require clinical studies based on ISO and IEC recognized standards to ensure safety and effectiveness, and in the future the FDA has outlined various scenarios in which clinical data will be necessary—e.g., when substantial equivalence between a new device and the chosen predicate(s) cannot be determined by non-clinical testing, such as animal, bench, or analytical testing. This scenario is similar to one in which a manufacturer has long-term market exclusivity for the clinical data, as competitors need to reproduce clinical evidence in order to show substantial equivalence under 510(k).

As an exemplary case study, the Apple ECG App (DEN180044) De Novo classification serves to illustrate how De Novo applicants can influence the requirements for clinical performance testing (special controls) needed to demonstrate the performance characteristics for follow-on 510(k) clearances. As noted in the DEN180044 Classification Order, to support their De Novo submission, Apple conducted a clinical study to establish a reasonable assurance of safety and effectiveness of the ECG App. Specifically, they conducted a prospective, parallel-cohort, multi-centre pivotal study using an enriched population of 602 subjects at 5 investigational sites. Three blinded independent board-certified cardiologists reviewed all the ECG recordings and assigned a classification. Their primary endpoint was the sensitivity and specificity of the ECG App algorithm in detecting atrial fibrillation (AF) compared with physician-adjudicated 12-lead ECG with performance goals of 90% and 92% respectively. For the secondary endpoint, the ECG app was required to produce a waveform with clinically equivalent information to the gold standard (Lead I ECG). The resulting 21 CFR 870.2345 (Product Code: QDA) section for electrocardiograph software for over-the-counter use requires special controls. These include: “Clinical performance testing under anticipated conditions of use must demonstrate the following: (i) The ability to obtain an electrocardiograph of sufficient quality for display and analysis; and (ii) The performance characteristics of the detection algorithm as reported by sensitivity and either specificity or positive predictive value.” Thus, while substantially equivalent follow-on devices can use the 510(k) clearance pathway with the Apple EECG App as a predicate, they still need to conduct a clinical study.

Since the De Novo applicant is the first mover, it can influence the extensiveness of the barriers to entry that follow-on products will face when submitting 510(k)s in its newly-established product code. This phenomenon may be amplified for data intensive SaMD products. For example, if the SaMD is a machine learning algorithm, the first mover with the largest dataset could set such a high bar for algorithm accuracy (or simply the size of the training/testing data) that it could make it more difficult for subsequent products to enter. Such a dynamic is unique to software products and highlights how a first entrant might be able to dominate a medical device product market.

Thus, the regulatory interaction of the De Novo classification process coupled with the subsequent 510(k) clearance pathway is ambiguous: on one hand, there are clear ways in which the barriers to market entry may be lower for follow-on innovators, but there are also clear opportunities for first movers to entrench their advantage vis-a-vis would-be follow-on entrants. Indeed, because both phenomena are likely to be stronger in the case of SaMD, a nuanced understanding of competition dynamics across different types of medical device software will be vital for ensuring both robust innovation incentives as well as competition in SaMD product markets.

### Are there unique IP considerations associated with the De Novo process that should be kept in mind by applicants and the FDA and do they differ for SaMD vs. hardware devices?

In addition to the potential for the De Novo process to impact competitive dynamics through special controls involving performance standards (e.g., required clinical studies), De Novo applicants may create additional barriers to entry by patenting core technological characteristics of their device, and tying the required performance standards to these key technological characteristics. Since follow-on 510(k) submissions using the original device as a predicate need to show “substantial equivalence,” this creates a risk for competitors of either implicitly admitting infringement in their 510(k) submissions, or not passing the test of “substantial equivalence” (NSE), thus failing to obtain market clearance^19^. Such a setting provides a significant opportunity for medical device innovators who possess patent protected technologies—especially those tied to the underlying technological characteristics of the device and likely to be used as part of the 510(k) substantial equivalence inquiry—to achieve sustainable competitive advantage for the duration of the patent term. Yet here too, the dynamics of medical device software lead to nuanced differences: since software patents are generally harder to enforce than hardware patents, such IP strategies may be less concerning for SaMD manufacturers.

In any case, the FDA should be mindful of this potential dynamic and ensure that the specific controls proposed by De Novo applicants are necessary to establish safety and effectiveness, and not an attempt to achieve competitive advantage via the interaction between the De Novo and 510(k) pathways with patent protection of core underlying technological characteristics^19^.

### What should we expect regarding future use of the De Novo pathway and what does this tell us about the potential role of the FDA in medical device innovation?

Following the possibility of “direct” De Novo requests created by FDASIA, the use of the De Novo *pathway* increased from 13 requests in 2012 to an all-time maximum of 44 requests in 2018 (Fig. 1). As noted, this peak immediately followed the FDA’s publication of regulatory guidance regarding the De Novo regulatory pathway.

Given the FDA guidance documents and increasing innovation in digital health and digital medical devices, one may expect that De Novo submissions will continue to steadily increase^15^. However, when considering future use of the De Novo pathway by medical device innovators it is important to note that the De Novo pathway is intended to create *new* medical device *categories*, as opposed to merely clear new devices. Upon a successful De Novo classification request, the Code of Federal Regulations (CFR) is updated to create a new regulatory category and product code for the novel *type of medical device*. We analysed all the De Novo device requests (*n* = 374) submitted to the FDA (1997 to August 2023) and the corresponding FDA decisions (De Novo Reclassification Orders). These have resulted in new product codes in 371 cases (99.2%). In effect, the De Novo process is primarily a regulatory pathway for: (1) *adding* categories of medical devices, and their associated new product codes and regulatory numbers to the CFR, (2) *classifying* the devices in these new categories according to their risk as either class I (low risk devices requiring general controls) or class II (moderate risk devices also requiring special controls), (3) *establishing* the first *predicate* device for the new product code (device category), and (4) *enriching the 510(k) pathway* by enabling follow on devices (with the same intended use and general technological characteristics) to use the 510(k) regulatory pathway for market clearance. Consequently, comparing the number of De Novo requests per year with the number of 510(k) submissions is misleading, as it compares new device *types* (categories, which can only be created once) to the typical “flow” of overall device clearances in existing product categories.

Once a new medical device category is created by the De Novo pathway, subsequent devices with the same intended use and general technological characteristics are reviewed and cleared through the 510(k) process. This helps explain, in part, why even for emerging technologies such as medical AI/ML the majority of the devices will be cleared through the 510(k) pathway^20^. Based on the regulatory interaction between the De Novo pathway and the 510(k), the number of De Novo requests would be expected to accelerate during periods of intense technological innovation and regulatory clarification, and subsequently stabilize (since similar follow-on devices would go through the 510(k) process based on the De Novo predicate) to the number of submissions representing the “steady state” of innovation of *categorically* different devices. In fact, a substantial increase in the number of successful De Novo requests would likely indicate a change in FDA practice and the allocation of CDRH resources—especially in the balance between the review of 510(k) submissions vs. De Novo classification requests. This may happen, for instance, if the FDA encourages De Novo requests to support the modernization of the current list of medical devices by adding both new device categories as well as newer predicates utilizing state-of-the-art digital technologies.

Barring changes in the way the FDA reviews 510(k) submissions and what it considers to be “substantially equivalent,” or further FDA guidance that especially promotes this pathway, we could expect the future use of the De Novo process to be in the range seen since 2017 (Fig. 1). For instance, there have been more AI/ML-enabled devices cleared through the 510(k) pathway than the De Novo classification requests^20^. This is expected for two reasons. First, a novel AI/ML-enabled device for which there is no 510(k) predicate results in a *single* De Novo classification, which then must be used as the predicate for subsequent substantially equivalent AI/ML devices. Second, the 510(k) determination currently does not take into account the nature of the algorithms. As noted earlier, a novel AI/ML device may still be “substantially equivalent” to a legacy 510(k) predicate based on older technology as long as the devices have the *same intended use* and *technological characteristics*. The FDA 501(k) review focuses primarily on hardware-related safety aspects including materials, energy use, and general principles of operation. Thus, if the new device shares these hardware characteristics with the older predicate, it may be cleared through the 510(k) pathway even though the algorithm is entirely different and medical AI-enabled. Given that a substantial amount of the novelty in modern medical devices lies on the algorithms that process the physiologic signals -as opposed to the hardware-, it would be advisable for the FDA to consider the nature of these algorithms (and their training sets) as part of their review of technological characteristics in order to determine whether differences between the algorithms in the devices raise “different questions of safety and effectiveness”.

## Discussion

The FDA’s regulatory and epistemic authority has three dimensions: directive, gatekeeping, and conceptual^21^. While the FDA’s legal authority is enshrined in statute, its epistemic authority manifests in scientific and technical standards that define what counts as valid evidence^22–25^. Our view is that the future use, success, and impact of the De Novo pathway is more likely to be influenced by how the FDA uses its “directive power” (i.e., the exercise of legal measures by the FDA over industry, especially through regulatory guidance mandates) as well as its use of “gatekeeper power” (e.g., setting the bar for substantial equivalence in 510(k) clearances) than by the underlying levels of medical device innovation.

Ideally, successful De Novo requests should capture innovation at the device category level (i.e., cases of product type innovation resulting in new product codes) while 510(k) clearances would capture the continuous-improvement innovations taking place at the device level (product market introductions for new medical devices in previously established categories through substantially equivalent intended uses and technologies). The current levels of activity indicate that ~1% of medical device innovations are at the category (product code) level, while 99% stem from continuous improvement in medical devices (i.e., just shy of 30 De Novo requests per year versus just shy of 3000 clearances through the 501(k) pathway per year).

In addition to medical device innovation at the *category* level, to a great extent, future use of the De Novo pathway will depend on what the FDA considers “substantially equivalent” as part of the 510(k) process. Given that most of the 510(k) device categories (product codes) and original predicates date back to 1976, the De Novo pathway provides an opportunity to update and modernize the list of regulatory device categories available to both manufacturers and regulators. However, if the FDA continues the practice of granting 510(k) clearances by stretching and broadly interpreting the original 1976 categories and predicates (e.g., clearing a modern digital health device for cough detection based on the medical magnetic tape recorder as a predicate [Product Code DHS], manufacturers will continue using the 510(k) process independently of the degree of novelty incorporated into the new medical devices, given that it is still, on average, a faster path to market). An exception would be sophisticated applicants with valuable IP (e.g., patent protection for key aspects of the underlying technology) and valuable clinical data at high evidence performance standards. These applicants may see the De Novo pathway as a strategic opportunity to (1) define an entirely new category of medical devices, (2) influence the special controls required for the category, including the need for performance standards requiring clinical studies to ensure safety and effectiveness, (3) establish their device as the *first predicate* in the category (and initially the sole device with clearance in that particular product code), and (4) potentially amplifying the value of their core patents by tying performance standards to these underlying technological characteristics. For example, if the SaMD is based on a machine learning algorithm, the first mover with a large proprietary dataset could set a high bar for algorithm accuracy (or size of the training/testing data) that it could significantly raise the barriers of entry for subsequent products to enter. For such medical device innovators the De Novo pathway presents a significant opportunity to gain sustainable competitive advantage, in part due to the interaction between regulatory realities (i.e., the joint operation of the *De-Novo* classification with the 510(k) clearance pathways) and IP (i.e., patent protection of core technological characteristics tied to performance standards).

The interaction of the digital transformation of the medical device industry and the availability of the De Novo pathway also presents new challenges and opportunities. Medical devices are becoming increasingly more complex, often including advanced biomedical signal processing algorithms and employing cloud-based computing. Relying on older predicates can be limiting when assessing the safety and effectiveness of the latest digital health technologies, as it often does not account for differences in the artificial intelligence/machine learning and signal processing algorithms which drive the performance standards, nor issues related to the device’s interconnectivity (e.g., cloud-based information security and data protection). As we have discussed here, the De Novo pathway holds significant potential for innovators seeking to define new categories of novel medical devices, as well as for the FDA to modernize the 510(k) pathway by promoting reliance on modern device predicates that represent a more accurate reflection of the state-of-the-art technology and current standards of safety and effectiveness.

## Methods

### Analysis of FDA De Novo classification orders and decision summaries

We analyzed all the De Novo device requests (*n* = 374) submitted to the FDA (1997 to August 2023) and the corresponding FDA decisions (De Novo Reclassification Orders).

The following primary data sources were used for analysis: (1) FDA Database “Device Classification Under Section 513(f)(2)(De Novo)”, (2) FDA Database “510(k) Premarket Notification (Clearances) under section 513(i)(1)(A) FD&C Act”, and (3) FDA Database “Premarket Approval”. Sources of evidence included the submitted De Novo request document and the corresponding *FDA* De Novo classification decisions (FDA Evaluation of Automatic Class III Designation De Novo Summaries), as well as the principal US federal laws (1938-2023) and the FDA regulations and guidance (1976–2023) relating to medical devices, including the (1) Food, Drug, and Cosmetic Act of 1938, (2) Radiation Control for Health an Safety Act of 1968, (3) Medical Device Amendments of 1976, (4) Safe Medical Devices Act of 1990, (5) FDA Modernization Act of 1997, (6) Medical Device User Fee and Modernization Act of 2002, (7) FDA Amendments Act of 2007, (8) FDA Safety and Innovation Act of 2012, (9) 21st Century Cures Act of 2016, and (10) FDA Reauthorization Act of 2017.

The FDA De Novo “Classification Orders” and “Decision Summaries” were reviewed to determine: (1) the prescription-only (Rx) vs over-the-counter (OTC) status, (2) the device type: Hardware, Software (SaMD) or In Vitro Diagnostic (IVD), and (3) the presence of algorithms (e.g., AI/ML) in the device.

## Data Availability

The data that support the findings of this study are available from the corresponding author upon request.

## Code availability

The code that support the findings of this study is available from the corresponding author upon request.
